# Application of β-Lactamase Reporter Fusions as an Indicator of Effector Protein Secretion during Infections with the Obligate Intracellular Pathogen *Chlamydia trachomatis*

**DOI:** 10.1371/journal.pone.0135295

**Published:** 2015-08-10

**Authors:** Konrad E. Mueller, Kenneth A. Fields

**Affiliations:** Department of Microbiology, Immunology & Molecular Genetics, University of Kentucky College of Medicine, Lexington, KY, United States of America; University of California, San Francisco, University of California, Berkeley, and the Children's Hospital Oakland Research Institute, UNITED STATES

## Abstract

*Chlamydia* spp. utilize multiple secretion systems, including the type III secretion system (T3SS), to deploy host-interactive effector proteins into infected host cells. Elucidation of secreted proteins has traditionally required ectopic expression in a surrogate T3SS followed by immunolocalization of endogenous candidate effectors to confirm secretion by chlamydiae. The ability to transform *Chlamydia* and achieve stable expression of recombinant gene products has enabled a more direct assessment of secretion. We adapted TEM-1 β-lactamase as a reporter system for assessment of chlamydial protein secretion. We provide evidence that this system facilitates visualization of secretion in the context of infection. Specifically, our findings provide definitive evidence that *C*. *trachomatis* CT695 is secreted during infection. Follow-up indirect immunofluorescence studies confirmed CT695 secretion and indicate that this effector can be secreted at multiple points during the chlamydial developmental cycle. Our results indicate that the BlaM-fusion reporter assay will allow efficacious identification of novel secreted proteins. Moreover, this approach can easily be adapted to enable more sophisticated studies of the secretion process in *Chlamydia*.

## Introduction

*Chlamydia trachomatis* (serovars D-K and lymphogranuloma venereum serovars L1-L3) are agents of human sexually transmitted disease, whereas ocular infections with *C*. *trachomatis* serovars A-C can lead to blindness [1]. *C*. *trachomatis* is a member of a larger *Chlamydiaceae* family that contains numerous species that have likely co-evolved with a eukaryotic host for >700 million years [2]. All *Chlamydia* spp. are Gram-negative obligate intracellular bacteria that possess a conserved, biphasic developmental cycle [3]. Development is initiated when infectious particles termed elementary bodies (EBs) invade host cells and differentiate into noninfectious, vegetative forms termed reticulate bodies (RBs). RB growth is eventually accompanied by asynchronous conversion of RBs to EBs. Subsequent exit from the host cell is mediated by lysis or extrusion [4]. Intracellular development occurs entirely within a parasitophorous vacuole termed an inclusion. Chlamydiae develop effectively segregated from the host cytosol since the inclusion membrane is passively impermeable to molecules >520 Da [5]. Despite this physical separation, *Chlamydia* spp. are capable of directly modulating host cell biology. Members of the *Chlamydiaceae* all express a type III secretion system (T3SS) to promote survival from within a protected niche.

Similar to systems in other T3S-expressing pathogens, the chlamydial T3SS is a multi-protein nanomachine capable of secreting and subsequently translocating (hereafter collectively referred to as secretion) anti-host proteins termed effectors, (T3SE) directly into an associated eukaryotic cell [6]. The chlamydial T3SS is present, and apparently active throughout development. EBs contain abundant levels of effectors required for invasion [7], and secretion can be detected within minutes of attachment to a host cell [8,9]. Described effectors first secreted during invasion include the translocated actin-recruiting phosphoprotein TarP [9], the translocated early phosphoprotein TepP [10], and the human Ahnak interacting protein designated CT694 [11]. Subsequent to entry, chlamydiae begin to secrete a number of identified effectors, the most abundant of which are the inclusion membrane (Inc) class of effectors. Many Inc effectors interact with elements of host vesicular transport pathways [12] while others likely play key roles in maintaining inclusion membrane architecture through interactions with other Incs [13,14]. The continued accumulation of Incs in the expanding inclusion membrane indicates that T3S is likely active in RBs until completion of the developmental cycle.

Interaction with T3S chaperones [10,15,16] and *in silico* analyses have been used successfully to identify chlamydial T3S substrates [17]. However, the use of surrogate T3SSs has been perhaps most efficacious in discovering putative chlamydial effectors. Several recent studies have leveraged *Yersinia* [18] or *Shigella* [19] T3SS in large-scale screens for T3S substrates. These studies and others have yielded a long list of potential effectors that require further validation. Confirmation of secretion by *Chlamydia* typically employs the use of indirect immunofluorescence assays to detect localization of the effector within or beyond the inclusion membrane [20]. This approach requires the generation of effector-specific antibodies. In addition, the assay can be confounded by low abundance of a given effector. Therefore, there is a need for an efficacious system to easily detect secretion of chlamydial proteins during infection.

Reproducible transformation of *Chlamydia* with a stably-maintained shuttle vector [21] has overcome a significant barrier that impeded efficient progress in investigating chlamydial infection biology. This approach has enabled development of second generation vectors expressing fluorescent proteins [22] or enabling conditional gene expression [23,24]. This approach has already been used to express epitope-tagged effectors [24,25] for efficacious detection of Inc secretion. Inc proteins have the advantage of being easily detectible due to concentration in the inclusion membrane. We regard it likely that detection of low-abundance, effectors that do not concentrate in a particular compartment will require signal amplification for detection. Numerous enzymatic tags have been employed in other T3SSs to demonstrate cytosolic localization of secreted effectors [26]. The use of β-lactamase (BlaM) translational fusions, originally developed to detect pathogenic *Escherichia coli* effector secretion [27], has proven to be a convenient and sensitive tool for detection of bacterial protein secretion. Infected cells are treated with the cell-permeant reagent CCF2-AM which is subsequently converted to a membrane-impermeant molecule by esterases in the host cytosol. CCF2-AM is composed of the flourophores coumarin and fluorescein. Excitation of intact CCF2-AM at 409 nm results in green fluorescent via fluorescence energy transfer (FRET). Secretion of a given bacterial protein-BlaM fusion into the host cytosol is readily indicated when CCF2-AM is cleaved by the BlaM moiety to disrupt FRET and yield blue fluorescence. This approach is particularly desirable for an obligate intracellular pathogen such as *Chlamydia* since evidence of secretion can be detected directly in the absence of host cell lysis. We therefore designed a two-step vector system that would enable ectopic expression of T3SE-β-lactamase chimeras. We provide proof-of-principle evidence herein that this system allows the robust detection of T3SE secretion in a tissue-culture infection model. We focused efforts on characterization of CT695. This putative T3SE is secreted by the *Yersinia* T3SS [11] and binds the chlamydial T3S chaperone Slc1[10,16], yet secretion by chlamydiae has not been confirmed. We reveal for the first time that *C*. *trachomatis* CT695 is secreted by chlamydiae at multiple stages of the developmental cycle.

## Methods

### Cell cultures and organisms

*C*. *trachomatis* serovar L2 (LGV 434) was cultivated in HeLa 229 epithelial cell monolayers (ATCC CCL-1.2; American Type Culture Collection, Manassas, VA), routinely maintained at 37°C in an atmosphere of 5% CO_2_/95% humidified air in RPMI-1640 containing 2 mM L-glutamine (GibcoLife Technologies Corporation, Grand Island, NY) supplemented with 10% (vol/vol) heat-inactivated fetal bovine serum (HIFBS; Sigma-Aldrich Company, St. Louis, MO). Where appropriate, intrinsically fluorescent chlamydiae were generated by labeling bacteria with CellTracker Red CMPTX (Life technologies) as described [28]. All EBs were purified from HeLa cells by centrifugation through MD-76R (diatrizoate meglumine and diatrizoate sodium injection U.S.P.; Mallingckrodt Pharmaceuticals, Mulhuddart, Ireland) density gradients (DG-purified) as previously described [29] and were used as the infection source for all experiments. Assessment of chlamydial growth was accomplished by enumeration of progeny EBs from 24 hr cultures as described [30]. Chemically competent *E*. *coli* 10-beta (NEB, Ipswich, MA) was used for routine cloning, and *dam* ^-^/*dcm* ^-^ *E*. *coli* (NEB) was used to propagate plasmids prior to transformation of chlamydiae. Where appropriate, 50 μg/ml carbenicillin was used for *E*. *coli* selection while 1.0 μg/ml cycloheximide and 0.6 μg/ml Penicillin G sodium (PenG) was used during chlamydial transformations.

### Vector construction

pGFP::SW2 was generously provided by Ian Clarke (University of Southampton). This template was modified using custom PCR primers (S1 Table; IDT, Coralville, IA). Sequence encoding mCherry was amplified from pmCherry-C1 vector using forward and reverse primers mC@GFP F and mC@GFP R, respectively. This was used to replace the GFP gene in pGFP::SW2 by insertion/deletion PCR as described [31] to produce pMC::SW2. Sequence encoding chloramphenicol drug resistance was amplified from pACD4K-C-loxP using forward and reverse primers SalI+AscI+Chlor F and SalI+AscI+Chlor R, respectively. In order to construct pL2dest, SalI restriction enzyme and Quick Ligation Kit (NEB) were used to digest and ligate the chloramphenicol drug resistance amplicon into pMC::SW2, simultaneously introducing AscI restriction sites around the chloramphenicol open reading frame. pUC19 was used as the backbone for construction of the β-lactamase translational fusions. The *Neisseria meningitidis* promoter was amplified from pGFP::SW2 and inserted into pUC19 by insertion/deletion PCR using forward and reverse primers NmP@puC F and NmP@pUC R, respectively, producing pUCNmP. *ct694-*, *ct695-*, *ct696-*, *euo-*, *groEL-*, and *tarp-bla* fusions were constructed by amplifying each open reading frame from *C*. *trachomatis* serovar L2 genomic DNA preparation, and by inserting each amplicon between the *Neisseria meningitidis* promoter and the full-length β-lactamase gene of pUCNmP by insertion/deletion PCR (primers listed in S1 Table). Each fusion was amplified from pUCNmP with primers NmP+BlaFus+AscI F and R, and inserted into pL2dest by AscI digestion followed by Quick Ligation (NEB). Loss of chloramphenicol resistance was used as a convenient marker for successful cloning of BlaM fusions in *E*. *coli*. Final constructs were transformed into *dam*-/*dcm*- *E*. *coli*, and plasmids were purified using a QIAfilter Plasmid Maxi Kit (Qiagen, Valencia, CA) prior to transformation into *C*. *trachomatis* L2. Q5 High-Fidelity DNA Polymerase (NEB) was used for all PCR amplifications and direct DNA sequencing (ACGT, Inc) was used to confirm all constructs.

### Chlamydial transformation

*C*. *trachomatis* L2 were transformed as described [21] with modifications. 2.6 x 10^6^ EBs and 2 μg of plasmid DNA were mixed in 50 μl CaCl_2_ buffer (10 mM Tris pH 7.4 and 50 mM CaCl_2_) and incubated at room temperature for 30 minutes. Each mixture was suspended in 2 ml Hanks Balanced Salt Solution (HBSS; Life Technologies) and applied to a 10-cm^2^ well containing a monolayer of confluent McCoy cells. Monolayers were infected by centrifugation at 900 x g for 1 hr at room temperature, after which HBSS was replaced with 2 ml RPMI + 10% FBS medium without drugs. At 7 hours post infection (hpi), cultures were supplemented with cycloheximide and PenG. Cells were harvested 48 hpi with a cell scraper and centrifuged at 20,000 x g for 30 min at 4°C. The pellet was suspended in 1 ml HBSS, centrifuged at 200 x g for 5 min at 4°C, and the supernatant was used to infect a new confluent monolayer of McCoy cells in a 10 cm^2^ well by centrifugation (900 x g, 1 hr, room temperature). Immediately after infection, medium containing both cycloheximide and PenG was added. Cells were harvested 48 hpi with a cell scraper, and the process of centrifugation and infection was repeated until transformed *Chlamydia* were recovered (typically 1 to 3 rounds of reinfection). In order to ensure clonal isolates, transformed *C*. *trachomatis* strains were diluted in HBSS and applied to confluent McCoy monolayers grown in 384-well plates (Greiner Cell Culture Microplate, catalog number 781091) at a concentration of one IFU for every 100 wells (approximately four inclusions per 384-well plate). Monolayers were infected by centrifugation with *C*. *trachomatis* at 900xg for 60 minutes at room temperature. Infection was allowed to continue in the absence of drug selection for seven days. Wells containing *C*. *trachomatis* (roughly one well for every 100) were then scraped with a p200 tip, and each isogenic population was then applied to new monolayers in T75 flasks for further expansion with antibiotic selection.

### Expression assays

For assessment of gene expression, RNA was harvested at indicated times using the Aurum Total RNA Mini Kit (Bio-Rad, Hercules, Ca.) according to the manufacturer’s instructions. RNA was converted to cDNA using the QuantiTect Reverse Transcription Kit (Qiagen). Transcript levels were determined by quantitative real-time PCR using the Bio-Rad CFX96 Real-Time Systen (Bio-Rad), iTaq Universal SYBR Green Supermix (Bio-Rad), and appropriate primers (S2 Table). All quantitative real-time PCR primers were confirmed to amplify with efficiencies >95%. For assessment of ectopically expressed protein levels, samples were obtained from HeLa cells infected with *C*. *trachomatis* at an MOI of 1. Cells were gently harvested with ice cold PBS and immediately concentrated by the addition of trichloroacetic acid to 10% (v/v) and centrifugation at 20,000 x g for 30 min at 4°C. Protein pellets were analyzed by SDS-PAGE electrophoresis followed by immunoblot analysis using β-lactamase-specific antibodies (Pierce, Rockford, IL) or α-Hsp60 (Santa Cruz, Dallas, TX) as a loading control. Proteins were visualized by peroxidase-conjugated secondary antibodies followed by development with ECL Prime (GE Healthcare, Pittsburgh, PA).

### BlaM Secretion Assay

The presence of fusion proteins in the cytosol of infected HeLa cells was observed directly with the use of the GeneBLAzer In Vivo Detection Kit (Invitrogen). HeLa monolayers were cultivated on glass cover slips to a confluence of ca. 75% and infected with *C*. *trachomatis* L2 expressing various β-lactamase-fusion proteins. CCF2-AM substrate was applied 24 hpi for 30 minutes, samples were fixed in 4% paraformaldehyde, and fluorescence was observed using a Leica TCS SP5 laser scanning confocal microscope. Images were processed equivalently using Adobe Photoshop CS2 version 9.0 (Adobe Systems, San Jose, CA).

### BSA-EGTA release assay

Cell-free release of secreted proteins from EBs was accomplished essentially as described [32]. Briefly, volumes of 5 x 10^7^ EBs were suspended in 50 mM acetate buffer and one replicate was supplemented with bovine serum albumin (BSA; Sigma) and EGTA pH 7.4 to 5 μM final concentration for each. EBs were incubated for 2 hrs at 37°C and bacteria were pelleted by centrifugation at 20,000 x g for 15 min. Proteins from bacterial pellets and cell-free supernatants were precipitated using trichloroacetic acid and subsequent pellets were suspended in equal volumes of SDS-PAGE solublization solution. Supernatant material was loaded at 5X bacterial pellets, proteins were resolved via SDS-PAGE, and probed in immunoblots with α-TarP [9], α-Hsp60 (Santa Cruz), and α-CT695 (described below). Proteins were visualized by peroxidase-conjugated secondary antibodies and chemiluminescence development.

### Fluorescence microscopy localization assays

Localization of CT695 and TarP was determined via indirect immunofluorescence using CT695-specific antibodies or α-TarP [9]. Full-length, His-tagged CT695 was used as antigen for production of antibodies. The coding sequence for *C*. *trachomatis* L2 CT695 was amplified using Q5 DNA polymerase and primers sets (5’-GGGGACAAGTTTGTACAAAAAA GCAGGCTTCAG TAGCATAAGCCCTATAGGGGGG-3’ and 5’-GGGGACCACTT TGTACAAGAAAGCTGG GTCCTATTAGATATTCCCAACCGAAGAAGG-3’) for transfer into the GATEWAY (Life Technologies) entry vector pDONR-221. Donor sequence was mobilized into pDEST-17 and constructs were verified via DNA sequencing (GENEWIZ). His-Tagged CT695 was expressed in *E*. *coli* BL21-Al (Invitrogen), and protein was purified to homogeneity via passage of lysates over TALON affinity resin (Clontech, Mountain View, CA). Polyclonal antibodies were raised in female New Zealand White rabbits as previously described [33]. To assess invasion-related secretion of endogenous CT695, HeLa cultures were infected for 1 hr with CMPTX-labeled *C*. *trachomatis* L2 at an MOI of ca. 10. Cultures were thoroughly washed and fixed for 20 min by treatment with 4% paraformaldehyde. Samples were permeablized by treatment with 0.1% Triton X100 in Tris-buffered saline supplemented with 5% BSA. *Chlamydia* were visualized via either intrinsic CMPTX label or with MOMP-specific antibodies [11,34]. All images were acquired by epifluorescence microscopy using a 60x apochromat objective plus 1.5x intermediate magnification on a TE2000U inverted photomicroscope (Nikon, Melville, NY) equipped with a Retiga EXi 1394, 12-bit monochrome CCD camera (QImaging, Surrey, BC, Canada) and MetaMorph imaging software version 6.3r2 (Molecular Devices, Downington, PA). Images were processed equivalently using Adobe Photoshop CS2 version 9.0 (Adobe Systems).

### Statistical Analysis

All presented data are representative of a minimum of three independent experiments. Quantitative data were generated from experiments containing triplicate biological replicates. All data are shown as mean of these replicates with 1 standard deviation. Calculation of standard deviation of the mean and assessment via Student’s t-test statistical analyses were performed using GraphPad Prism 6 version 6.04 (Graphpad Software Inc., La Jolla, CA).

## Results

**Fig 1:**
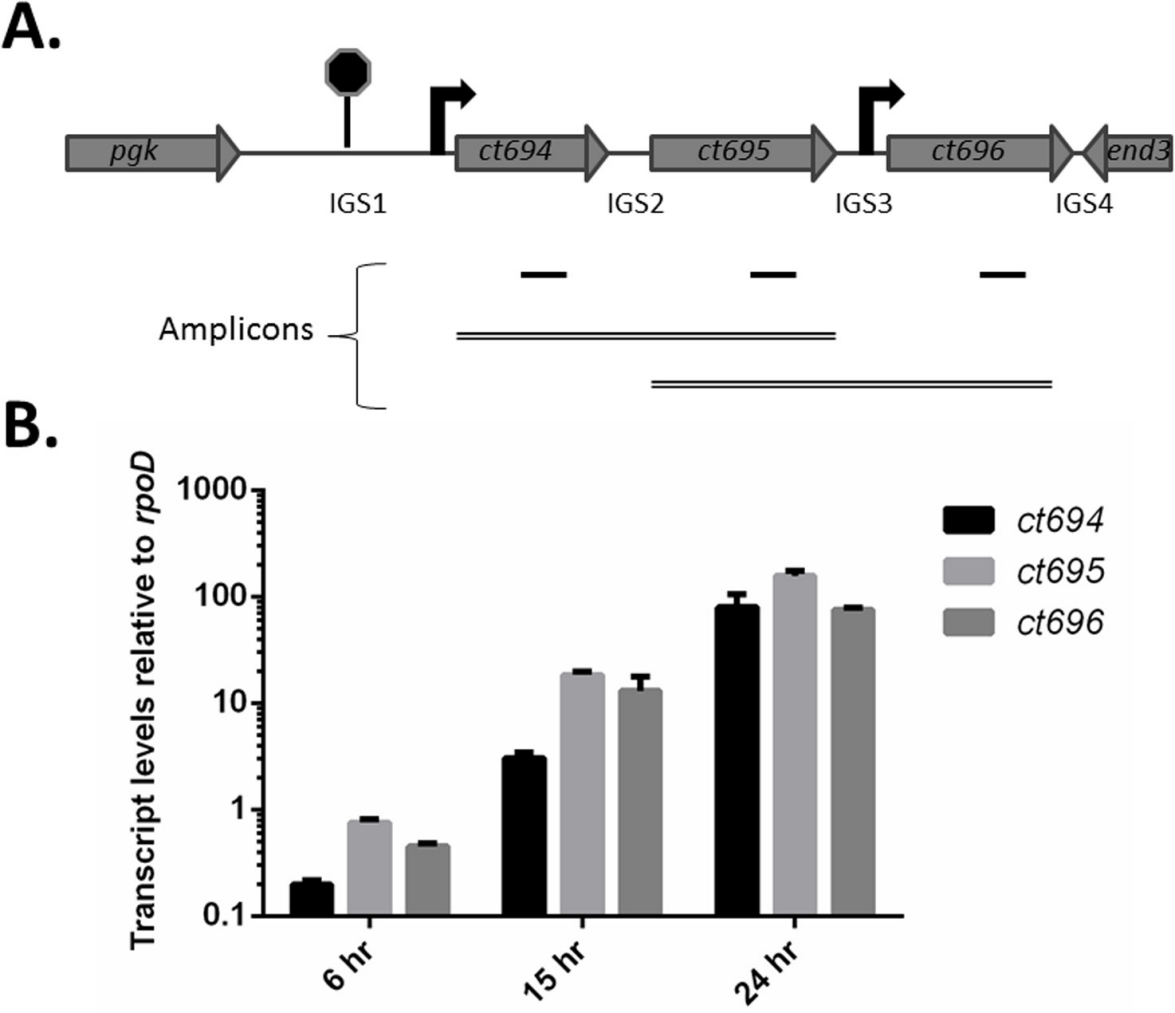
Gene arrangement and relative expression of *ct694*, *ct695*, and *ct696*. (A). Schematic arrangement of the *ct694*-*ct696*. The locus is flanked by phosphoglycerate kinase (*pgk)* and endonuclease III (*end3*) genes and contains 4 intergenic regions (IGS1-4) of 261, 48, 54, and 3 nucleotides, respectively. TransTermHP [36] predicts a Rho-independent transcriptional terminator between *pgk* and *ct694*. Arrows indicate relative positions of previously reported transcriptional start sites [38]. Amplicons used for expression analyses are represented schematically. 100 bp amplicons (solid lines) were generated for qRT-PCR wherease gene-spanning amplicons (double lines) were employed to test the possibility of polycistronic message. (B). Transcription of *ct694*, *ct695*, and *ct696* increases throughout chlamydial development. HeLa cells were infected with *C*. *trachomatis* L2 at an MOI of 0.5, and transcript levels were determined by quantitative real-time PCR at various time points throughout the chlamydial developmental life cycle. Expression levels were normalized against, and relative to, those for the constitutively expressed *rpoD*.

**Fig 2:**
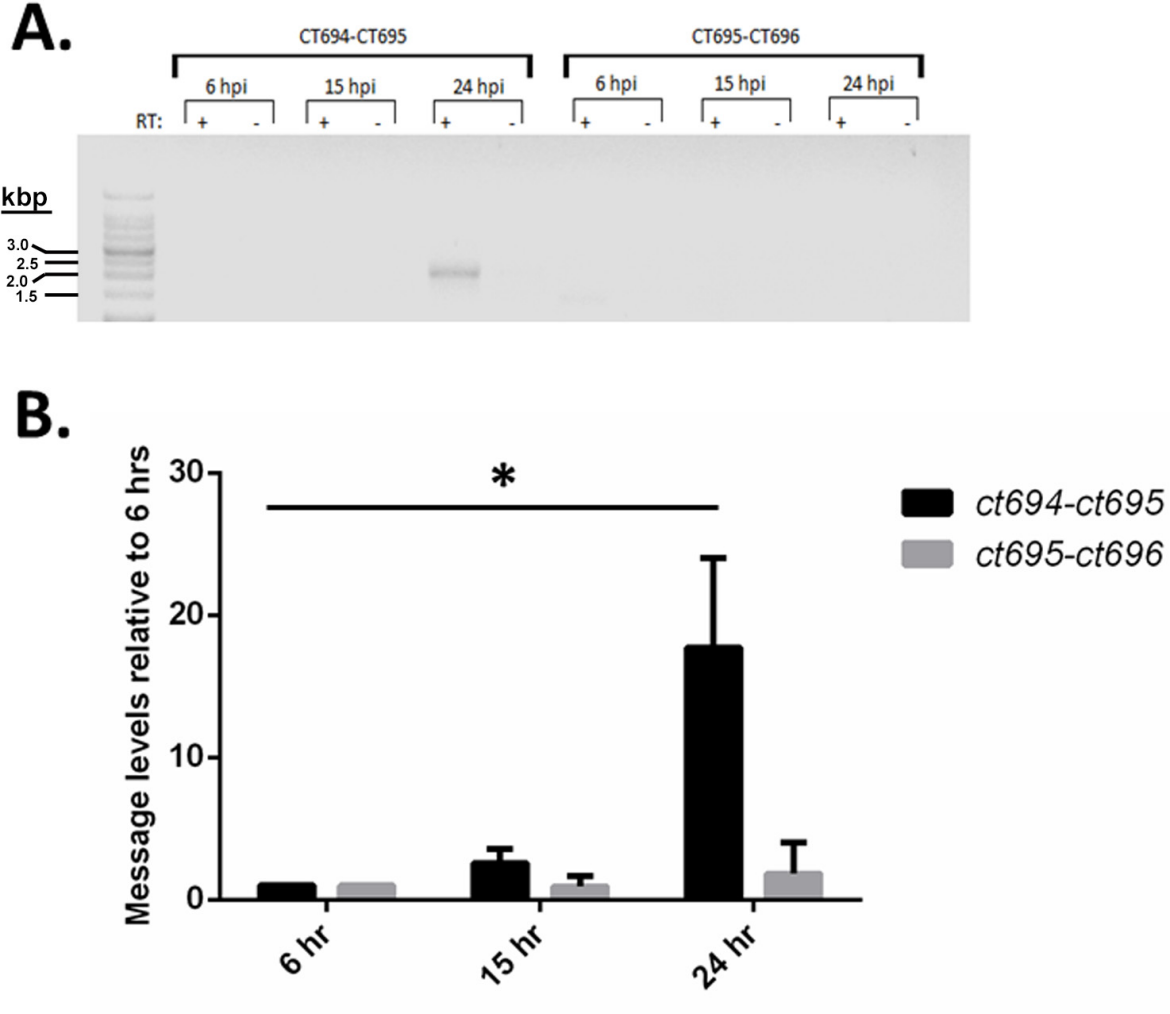
Co-transcription of *ct694* and *ct695*. (A). The presence of transcripts containing multiple open reading frames was determined by reverse-transcription (RT) PCR with primers surrounding *ct694* and *ct695* or *ct695* and *ct696*. RNA was isolated from HeLa cells infected with *C*. *trachomatis* L2 at an MOI of 0.5 grown to various time points post infection. (B). The same samples were additionally analyzed by quantitative real-time PCR for increased sensitivity. Levels shown are relative to those detected 6 hpi. A Student’s T test with Welch’s correction was employed to assess statistical significance (*, P < 0.04).

We began by assessing whether CT695 is encoded within a dedicated T3S-related locus. *C*. *trachomatis* CT695 is encoded [35] immediately downstream from the validated T3SE CT694 and upstream from the hypothetical CT696 (Fig 1A). Flanking gene orientation and a predicted rho-independent transcription terminator upstream from *ct694* [36] indicate that *ct694*, *ct695*, and *ct696* could comprise an operon. Transcriptional profiling of *C*. *trachomatis* serovar D via microarray indicated that *ct694* is expressed much later than *ct695* or *ct696* [37]. However, deep-sequencing-based transcriptome analysis of *C*. *trachomatis* L2 revealed a common transcriptional start site for *ct694* and *ct695* [38], raising the possibility of polycistronic message. We assayed temporal gene transcription by RT-PCR from total culture RNA harvested from *C*. *trachomatis* L2 infected HeLa cells 6, 15, or 24 hpi. Samples were normalized to *rpoD* and transcript levels were assayed for *ct694*, *ct695*, and *ct696* (Fig 1B). Basal levels of message were detected for all three genes as early as 6 hpi. As expected, these levels were increased (ca. 10-fold) at each of the later time-points. We next addressed transcriptional linkage using the previously reported approach [39] using RT-PCR and gene-spanning primer sets. A correctly sized amplicon (2.2 kb) was detectible only in the presence of RT and *ct694*-*ct695* spanning primers at 24 hpi (Fig 2A). No product was detected using *ct695*-*ct696* spanning primers even though these primers were capable of yielding the 2.4 kb product using DNA template (data not shown). A faint band was detected in the 6 hpi sample, but the apparent size was below 1.5 kb. Since qRT-PCR indicate the presence of message at earlier time-points, we used gene-spanning primers and qRT-PCR as a more sensitive means to detect polycistronic message (Fig 2B). In agreement with the RT-PCR data, an amplicon containing *ct694* and *ct695* was apparent at 24 hpi. Although a small (2.75-fold) increase over background was detected for *ct694*/*ct695* at 15 hpi, this was not statistically significant. No product was detected for *ct695-ct696*. Taken together, these data are consistent with mid-cycle expression of *ct694*, *ct695*, and *ct696*. However, *ct694* and *ct695* are likely transcribed separately from *ct696*, and *ct694*/*ct695* expression at 15 hpi is likely due to individual promoters and not co-transcription.

**Fig 3:**
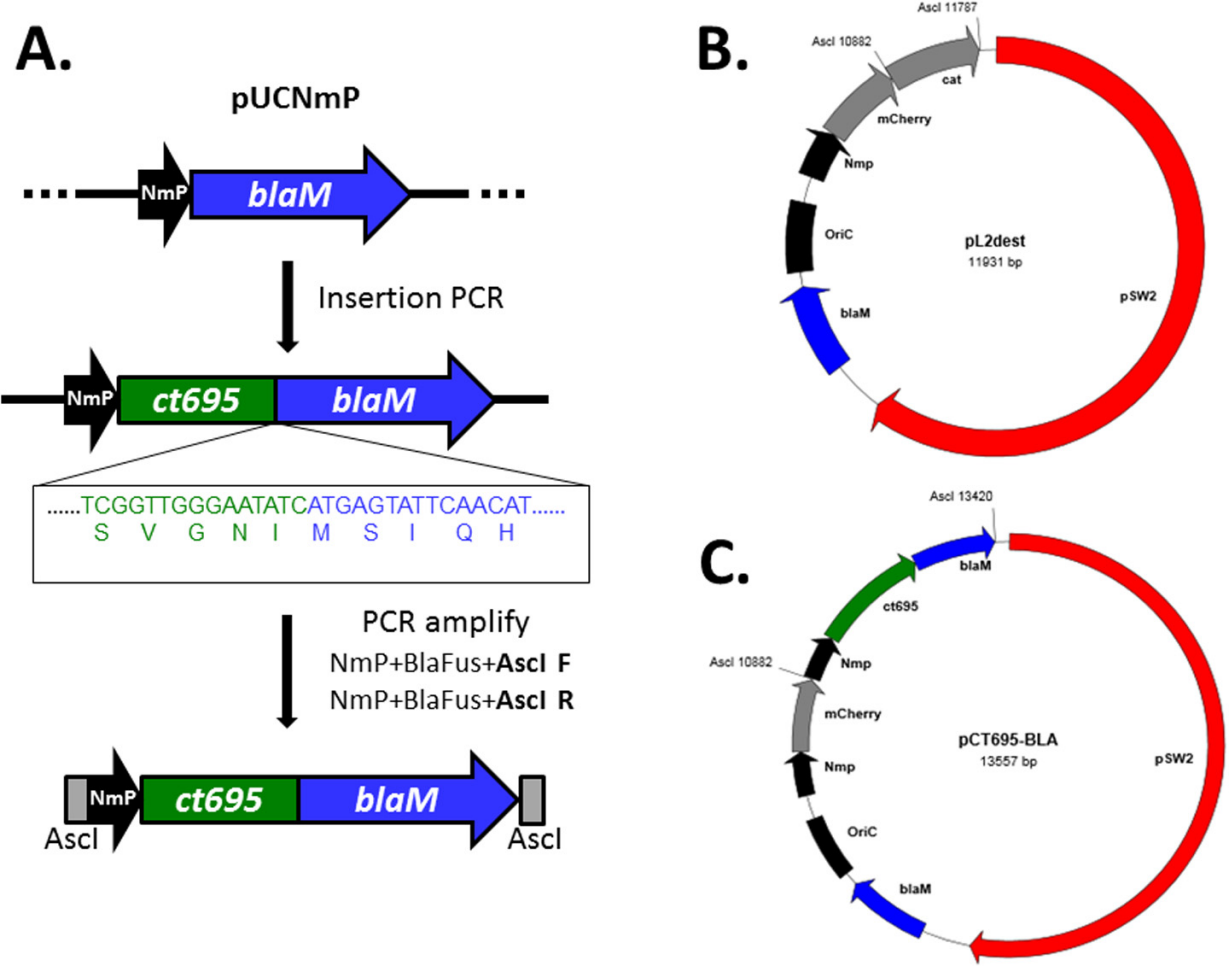
A schematic of constructed plasmids using CT695 as an example. (A). pUCNmP. The *Neisseria meningitidis* promoter (NmP) was inserted into pUC19 upstream from BlaM. Insertion/Deletion PCR is used to insert any chlamydial sequence (*ct695* is shown) to create a translational fusion of the chlamydial gene (green) with the β-lactamase gene (blue). DNA elements can then be PCR amplified using primers NmP+BlaFus+AscI F and NmP+BlaFus+AscI R to generate a product flanked by AscI restriction sites. (B). pL2dest was created by replacement of the coding sequence for GFP/CAT of pGFP::SW2 with the mCherry gene. A chloramphenicol drug cassette flanked by AscI recognition sequences was introduced immediately downstream from mCherry coding sequence. (C). pCT659-BLA was created by ligation of AscI-digested PCR product into the AscI site in pL2dest. The resulting plasmid allows expression of CT695-Bla from the constitutive Nmp promoter.

Transcriptional linkage of *ct694* and *ct695*, coupled with previously reported secretion by the heterologous T3SS [11] and association with the chaperone Slc1 [10,16], predicts that CT695 is secreted by chlamydiae. We wanted to take advantage of the newly acquired ability to transform *Chlamydia* in order to construct a reporter system that would facilitate assessment of protein secretion during infection (Fig 3). The entry vector pUCNmP contains the *N*. *meningitidis* promoter (NmP) described by Wang, et al. [21] positioned upstream from the complete TEM-1 β-lactamase coding sequence. This enables insertion of any chlamydial gene using insertion PCR [31] to create an in-frame fusion with BlaM. PCR primers flanked with the AscI recognition-site sequence are then used to amplify the construct, followed by digestion and ligation with pL2dest, a derivative of pGFP::SW2 [21] with GFP in place of mCherry and an engineered AscI restriction site.

**Fig 4:**
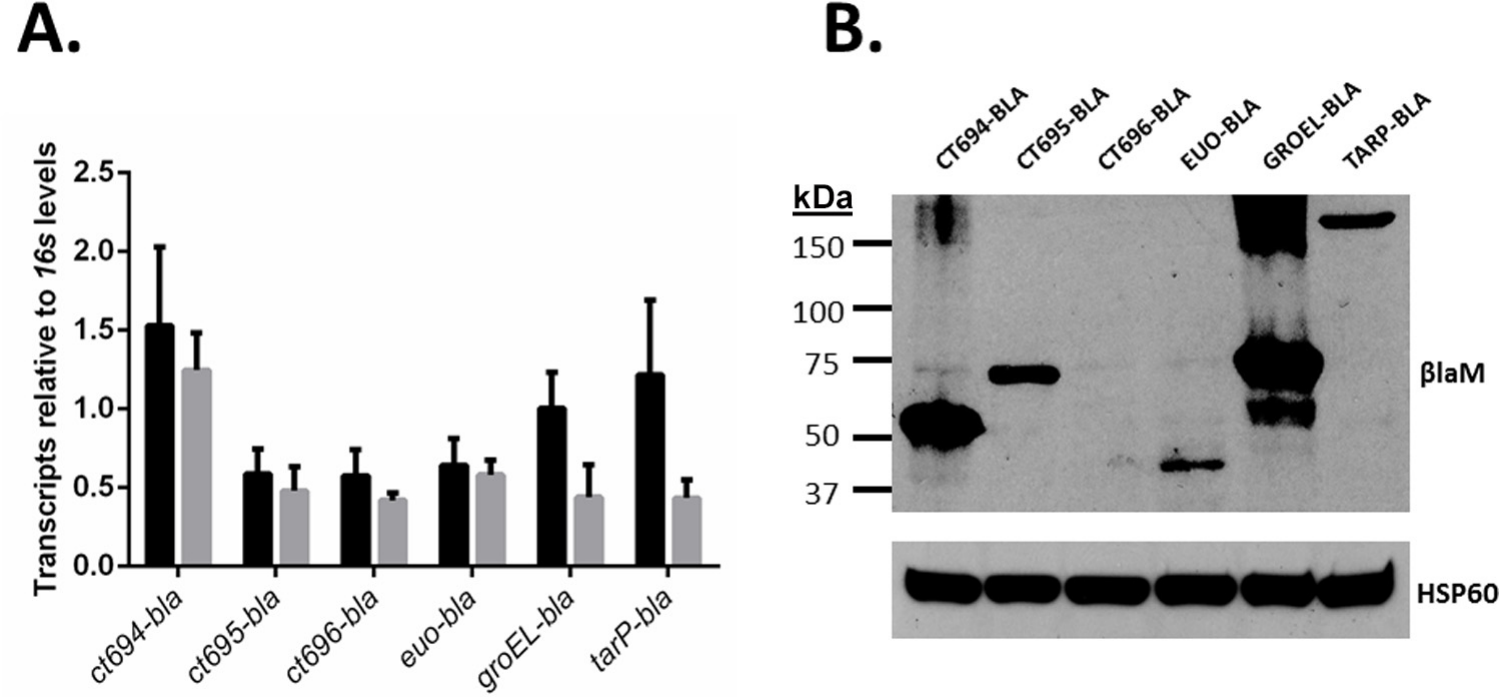
Expression of fusion constructs in *C*. *trachomatis* L2. (A). Transcription of *blaM* fusions (grey bars) and *mCherry* (black bars) was measured by reverse transcription followed by quantitative real-time PCR. Levels were normalized to those of chlamydial *16s*. Total RNA was isolated 24 hpi from HeLa cells infected with transformed *C*. *trachomatis* L2 at an MOI of 1. (B). Total protein was isolated 24 hpi from HeLa cells infected with transformed *C*. *trachomatis* L2 at an MOI of 1, and samples were probed with BlaM- or chlamydial Hsp60-specific monoclonal antibodies. Size standards are indicated in kDa.

The coding sequences for CT694, CT695, and CT696 were PCR-amplified from *C*. *trachomatis* L2 and mobilized into pUCNmP to create translational fusions with the downstream βlaM gene. Similar constructs containing Tarp and Euo or GroEL were generated as positive and negative secretion controls, respectively. Entry clones were subsequently transferred into pL2-dest and used to transform *C*. *trachomatis* L2. PenG selection was applied, and transformed chlamydiae were isolated by limiting dilution. None of the constructs appeared to impact chlamydial growth since progeny IFUs were unchanged at 24 hpi compared to untransformed chlamydiae (S1 Fig). HeLa cells were infected with respective strains and whole culture RNA or protein was isolated at 24 hpi to assess for trans-gene expression. qRT-PCR of total RNA was employed to assess respective transcript levels in comparison to mCherry message (Fig 4A). Similar levels of each message were detected, consistent with the use of a constitutive Nm-promoter. While mCherry transcript appeared more abundant that *groEL-bla* and *tarp-bla*, these differences were not statistically significant. Whole-culture proteins were probed in immunoblots with BlaM-specific antibodies to visualize individual chimeric proteins and Hsp60-specific antibodies as a loading control (Fig 4B). Both CT694-BLA and GroEL-BLA were detected in abundant amounts. Euo-BLA, TarP-BLA, and CT695-BLA were readily detectible but at comparably lower amounts. We were unable to detect CT696-BLA despite multiple attempts. With the exception of CT696, we therefore reasoned that chimeric proteins were expressed at sufficient levels to progress to secretion assays in *Chlamydia*-infected cells.

**Fig 5:**
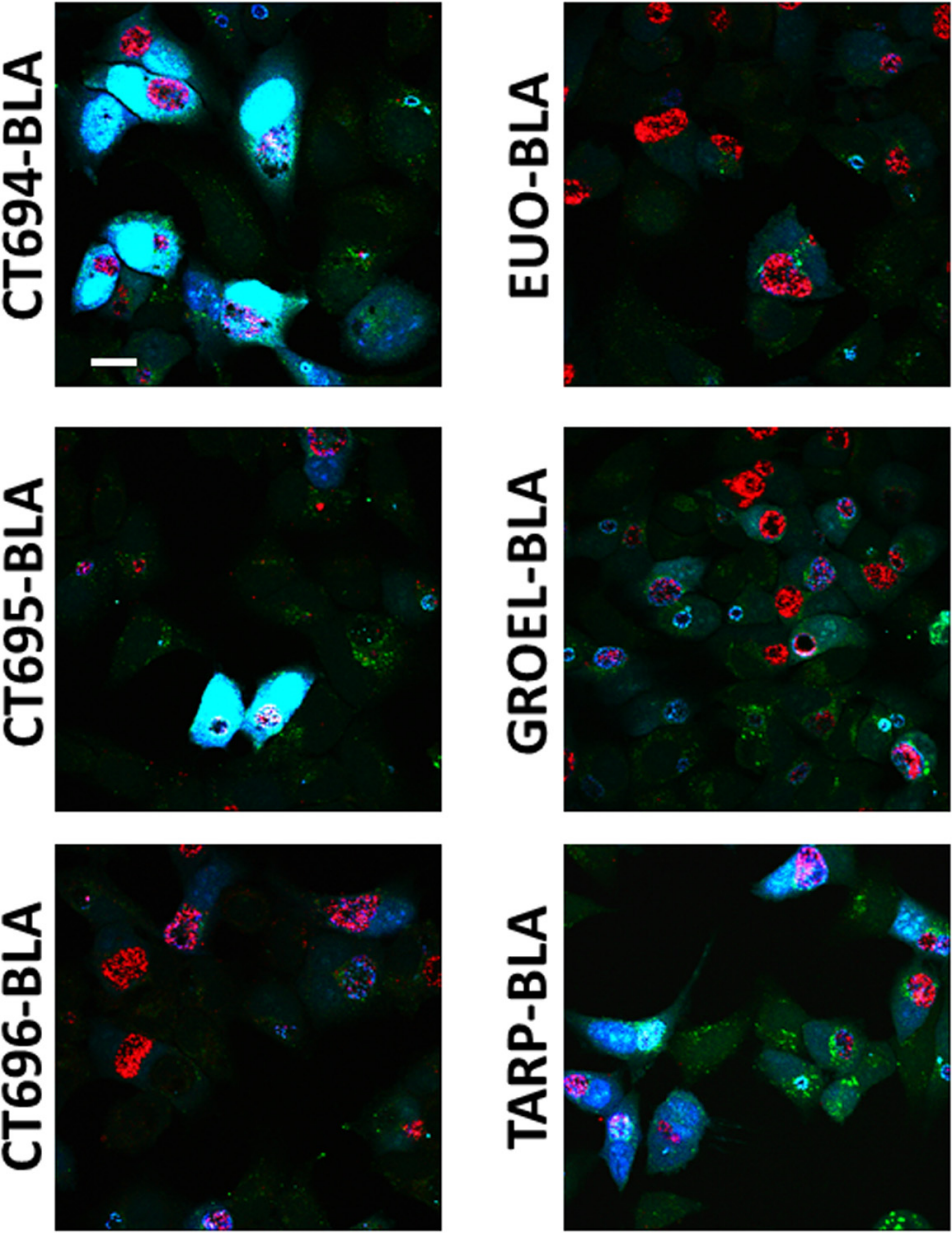
Detection of BlaM fusions in the cytosol of the host cell. 24 hpi, HeLa cells infected with transformed *C*. *trachomatis* L2 were examined for the presence of cytosolic BlaM with the GeneBLAzer Detection Kit. Samples were treated with CCF2-AM for 30 minutes, fixed with 4% paraformaldehyde, and examined by confocal microscopy. Chlamydial expression of CT694-, CT695-, and TarP-BlaM constructs produced significant blue-fluorescent signal in the cytosol of infected cells. Bar = 10 μm.

We utilized the established [26] FRET disruption via BlaM-mediated cleavage of CCF2-AM to test the ability of chimeric proteins to gain access to the host cytosol. HeLa cells were infected for 24 hrs and then loaded with CCF2-AM for 30 min. Cultures were paraformaldehyde fixed and processed for confocal immunofluorescence (Fig 5). In each case, chlamydial inclusions were visualized by mCherry-derived signal. As expected, mature chlamydial inclusions were detectible in each expression strain. Disruption of FRET results in blue signal, and robust signal was detected in HeLa cytosols when CT694-BLA, CT695-BLA, or the positive control TarP-BLA were expressed by chlamydiae. Only green fluorescence—indicative of intact CCF2-AM—was prominently detected when either GroEL-BLA or Euo-BLA were expressed. The apparent lack of blue signal in the presence of CT696-BLA was inconclusive given our inability to detect this chimeric protein. We did not detect significant green or blue signal within the inclusion lumen or within bacteria. Hence it is likely that eukaryotic cytosolic esterases cleave CCF2-AM to preclude passage across the inclusion membrane. These data indicate that the BlaM reporter system is applicable during *Chlamydia* infection and indicate that, similar to CT694 and TarP, CT695 is a secreted chlamydial protein.

**Fig 6:**
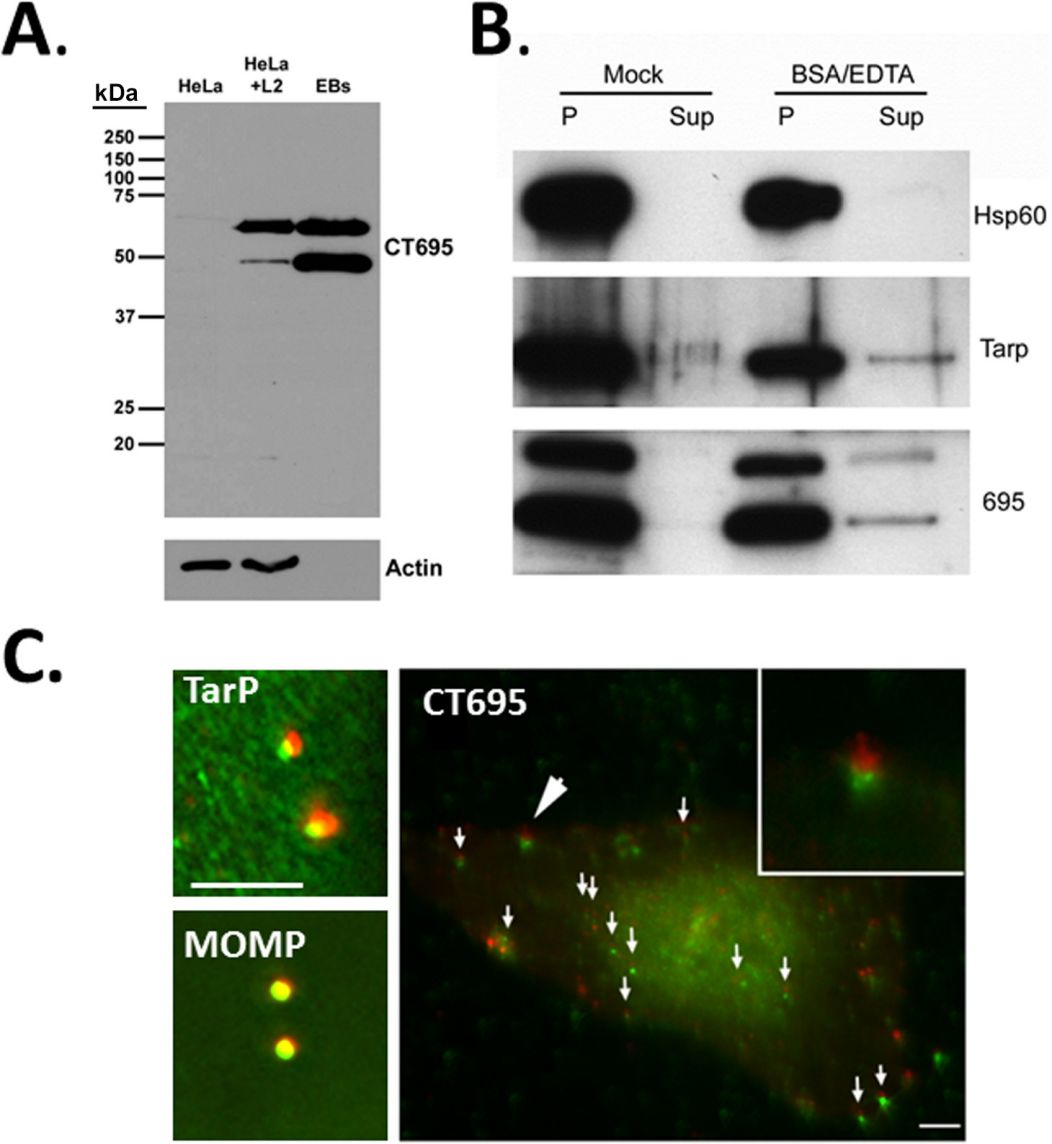
CT695 is secreted by EBs during invasion. (A). CT695-specific antibodies were used to probe lysates of *Chlamydia*-infected culture lysates (HeLa + L2) or lysates of purified *C*. *trachomatis* L2 EBs (EB). Lysates of corresponding uninfected HeLa cells (HeLa) were added as a negative control and all lysates were probed with α-actin as a loading control for whole-culture material. (B). Purified preparations of *C*. *trachomatis* L2 EBs were mock treated or treated with BSA and EDTA. Samples were subsequently centrifuged and material from cell-free supernatants (Sup) and chlamydiae-containing pellets (P) were probed in immunoblots with Hsp60, TarP, or CT695-specific antibodies. All proteins were visualized with HRP-conjugated secondary antibodies and subsequent chemiluminescence development. (C). HeLa cells were infected with CMPTX-labelled *C*. *trachomatis* L2 at an MOI of 10 and fixed paraformaldehyde fixed at 1 hpi. Cells were probed with TarP-, MOMP- or CT695-specific antibodies. Chlamydiae are shown in red while CT695, MOMP, and TarP localization was visualized with secondary antibodies conjugated to Alexa-488 (green). Merged channels of epifluorscence images are shown. Large arrow designates area of inset and small arrows signify other areas where CT695 appears as punctate signal adjacent to EBs. Scale bar = 5 μm.

While the BlaM reporter indicates secretion, this approach does not indicate specific protein localization. We generated antibodies specific for full-length CT695 to examine CT695 immunolocalization and confirm secretion of endogenous protein. Antiserum specificity was confirmed by probing lysates of uninfected HeLa cells, *C*. *trachomatis*-infected HeLa cells or density-gradient-purified *C*. *trachomatis* EBs (Fig 6A). Interestingly, CT695 migrated as a doublet with the lower band being most abundant in EB lysates. Despite a faint cross-reactive band apparent in HeLa lysates, these antibodies appeared specific and were used in subsequent analyses. TarP is packaged in EBs and low levels of this effector can be released by treatment of purified EBs with BSA and EDTA [32,40]. To test whether CT695 behaved similarly, EBs were mock-treated or treated with BSA/EDTA followed by centrifugation to generate an EB-containing pellet and a cell-free supernatant (Fig 6B). These fractions were probed in immunoblots with our CT695-specific antibodies. Material was probed with α-Tarp as a positive control or α-Hsp60 as a negative control. All proteins were detected in EB pellets, and CT695 reproducibly migrated as a doublet. Similarly to TarP, CT695 was detected in the cell-free supernatant only after treatment with BSA and EDTA. Hsp60 was not released from EBs by this treatment. We next asked whether immunolocalization of CT695 resembled that reported for TarP and CT694 during infection [11]. HeLa cells were infected with intrinsically-labeled *C*. *trachomatis* L2 at an MOI of ca. 10 for 1 hr, fixed with paraformaldehyde, and processed for analysis via epifluorscence (Fig 6C). Red-labeled EBs marked the position of chlamydiae while MOMP, TarP, and CT695 were detected using antibodies (green). While typical background staining was visible, both TarP- and CT695-specific signal was typically detected concentrated immediately adjacent to EBs in a pattern consistent with secretion of these proteins. In contrast, MOMP-specific signal overlapped with red EBs. Taken together, these data indicate that EBs are loaded with CT695, and this pool of protein can be secreted during the invasion process.

**Fig 7:**
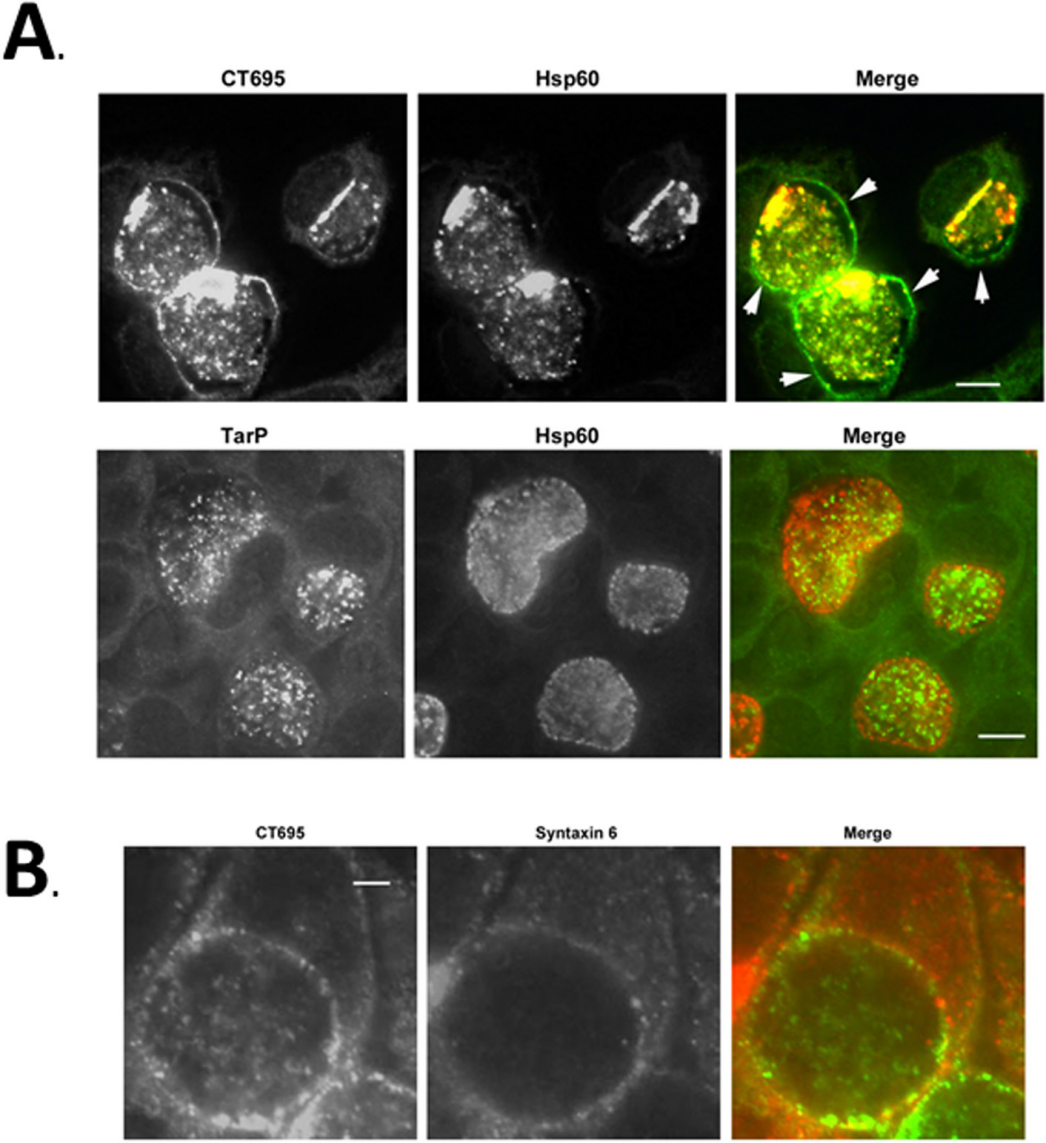
CT695 is secreted during late-cycle development and co-localizes with the chlamydial inclusion. HeLa cells were infected with *C*. *trachomatis* L2 at an MOI of 1 and paraformaldehyde fixed at 24 hpi. (A). CT695 or TarP localization were assessed using α-CT695 or α-TarP, respectively. Chlamydiae were detected using α-Hsp60. Individual and merged channels of epifluorscence images are shown with specific detection of *Chlamydia* (red) and CT695 or TarP (green). Arrows indicate apparent inclusion membrane localization and scale bar = 10 μm. (B) HeLa cells were infected with *C*. *trachomatis* L2 at an MOI of 1 and paraformaldehyde fixed at 24 hpi. CT695 was detected with α-CT695 (green) wherease the position of the chlamydial inclusion membrane was visualized via staining with antibodies specific for Syntaxin-6 (red). Scale bar = 5 μm.

Finally, we extended our analysis to test localization of CT695 during a later stage of development. HeLa cells were infected with *C*. *trachomatis* L2 and processed for immunofluorescence microscopy at 24 hpi (Fig 7A). Staining with α-CT695 revealed signal that co-localized with Hsp60-stained chlamydiae as well as in a rim-like staining pattern typical of inclusion membrane staining. In contrast, TarP-specific staining was confined to intra-inclusion chlamydiae. The pattern was consistent with staining of EBs since inclusion membrane-localized RBs seemed to lack significant staining with α-TarP. TarP staining is comparable to CT694 which can only be detected co-localizing with bacteria at this time-point [11,41]. Host syntaxin 6 has been previously shown to associate with the *C*. *trachomatis* inclusion membrane [42]. We therefore stained inclusions with syntaxin-6 and CT695-specific antibodies to confirm inclusion membrane association of CT695 (Fig 7B). The rim-like pattern of CT695 signal overlapped with syntaxin 6-specific signal, indicating that CT695 likely accumulates in the host cytosol adjacent to the inclusion membrane. These data indicate that CT695 is secreted at later stages of development where it can associate with the inclusion membrane.

## Discussion

Prior to development of techniques to genetically manipulate chlamydiae, detection of protein secretion during infection traditionally involved the use of specific antibodies to examine protein localization via indirect immunofluorescence. Detection of the putative effector in the inclusion membrane or extra-inclusion spaces represented the sole indicator that a protein was secreted by chlamydiae. The recently acquired ability to reproducibly transform *Chlamydia* with a stably-maintained shuttle vector [21] has opened the door to more efficacious approaches to directly test for protein secretion. For example, ectopic expression of epitope-tagged Inc proteins has surmounted the need to generate antigen-specific antibodies for a putative secreted protein [24,25]. This approach was also used to confirm secretion of the type II secretion substrate CPAF [24]. These data suggest that effector-reporter fusion proteins represent a useful approach for examination of protein secretion in a tissue-culture infection model.

Fusion of TEM-1 β-lactamase to secretion substrates was originally developed to examine T3SE secretion in pathogenic *E*. *coli* [27] and has become widely used to study secretion by extracellular and facultative-intracellular bacteria [26]. We wondered whether this approach could be adapted to an obligate intracellular pathogen such as *Chlamydia*. In support of this concept, BlaM fusions have been used successfully to examine protein secretion by *Coxiella burnetti* [43–45]. We chose to focus initial proof-of-principle experiments on the locus containing *ct694-ct696*. CT694 is a validated T3SE that is secreted during the invasion process and interacts with host cell AHNAK [11]. We thought it likely that CT695 could also be secreted by chlamydiae since this protein can be secreted by the heterologous *Yersinia* T3SS [11]. In addition, CT695 shares a common transcriptional start site [38] and chaperone [10] with CT694. We did not detect secretion of CT696 in *Yersinia* [11], yet de Cunha, et al [18] reported indeterminate results leaving open the possibility of low-level secretion of CT696 by yersiniae.

Hence this locus was ideal for the development of our chlamydial secretion reporter system since it contained a *bona fide* secretion substrate, a putative secretion substrate, and a questionable substrate.

To accomplish our goal, we generated a two-vector system to first enable general construction of BlaM fusions with any chlamydial gene downstream from a constitutive *N*. *meningitidis* promoter. We chose the constitutive Nmp promoter since it has been functionally validated in *Chlamydia* [21] and to avoid any regulatory complications that could arise from ectopic-overexpression from endogenous promoters. Standard primer sets enable amplification and subsequent AscI-mediated cloning into a *Chlamydia* shuttle vector. Transformation of *Chlamydia* is selected for by resistance to PenG. Constitutive expression of mCherry serves as an easily identifiable indication of transformation and as an internal control for normalization of gene expression. Genes encoding CT694, CT695, and CT696 were mobilized into *Chlamydia* using this system. We also included the T3SE TarP as a positive control and two non-secreted proteins, Euo and GroEL, as negative controls. The presence of neither vector-only nor chlamydial genes interfered with chlamydial development. This was true for Euo, which has been shown to repress gene transcription [46,47]. However, we anticipate that this result is likely specific to the chlamydial gene being expressed. We recommend that growth be directly assessed for each new construct since we cannot rule out the possibility that ectopic expression of other chlamydial genes could alter chlamydial development. Ectopic expression of genes in the presence of endogenous, genomic copies of respective genes was another potential complication since competition for the secretion apparatus or chaperones could have interfered with secretion. In agreement with previous studies overexpressing IncD [24,25], this did not appear to be a factor. Indeed, trans-encoded overexpression of T3SE is routine in other T3SS-expressing bacteria. Although Tarp, CT694, and CT695 share Slc1 as a secretion chaperone [10], most T3SE contain both chaperone-dependent and independent secretion signals [48]. Therefore, it is perhaps not surprising that overexpression of secretion substrates in *Chlamydia* is a productive approach. We cannot exclude the possibility, however, that competition among recombinant and endogenous proteins could result in false negative results in this assay.

With the exception of CT696, all constructs were well expressed in *Chlamydia*. Although *ct696-bla* transcript was produced at levels comparable to the other constructs, protein levels were below detectable limits. The precise cause of this is unclear. However, we recognize the possibility that certain proteins which are natively expressed at extremely low levels may lack the translational machinery to allow for expression of additional constructs regardless of transcript levels. This result highlights the possibility that ectopic expression may not be possible for all chlamydial gene products. Regardless, results for the remaining constructs were conclusive. BlaM fusion to CT694, CT695, and TarP all resulted in blue signal indicative of cytosolic CCF2-AM cleavage. Hence, these proteins were clearly secreted into the host cytosol. Our vector contains an additional vector-encoded *blaM* conferring penicillin resistance, that could have confounded results. For example, chlamydial lysis in conjunction with an unexpectedly permeable inclusion membrane could have led to spurious BlaM in the HeLa cytosol. However, Euo and especially the abundant GroEL BlaM fusions did not yield significant blue signal. In addition, an extra copy of BlaM did not confound a similar approach in *C*. *burnetti* [43]. Finally, strains have been passaged at least 8 times without loss of the intact plasmid (data not shown). Therefore recombination among multiple gene copies does not seem to be an issue. We currently have no means to confirm that secretion by *Chlamydia* is dependent on the T3SS. Any genetic lesion rendering T3S inactive is likely to be lethal to the bacteria. Although chemical inhibitors of type III secretion such as salicylidene acylhydrazides have been employed [49], they appear to not specifically target T3SS [50,51]. Based on secretion in heterologous T3SS [11] we can only infer this pathway for deployment.

Evidence for secretion of TarP [9] and CT694 [11,41] has been restricted to invasion. Our results are clearly consistent with continued secretion of TarP- and CT694-containing fusion proteins later in development. Whether this finding reflects temporal secretion patterns for endogenous proteins remains unclear. The T3SS is clearly active throughout chlamydial development [52], and it is possible that forced expression of TarP and CT694 could result in atypical timing for secretion. However, we were able to detect endogenous CT695 at later times since the protein was concentrated at the inclusion membrane. Since CT694 and CT695 can be transcriptionally linked, it is plausible that CT694 is also secreted during later development. Although immunoblot revealed detectible levels of CT694 throughout development [11], detection of endogenous protein via immunolocalization was likely confounded by low abundance and/or the lack of effector concentration in a specific cellular compartment.

How CT695 may be contributing to chlamydial infection remains to be determined. We detected evidence of endogenous CT695 secretion during invasion and subsequent development. We conclude that, similar to TarP, TepP, and CT694, CT695 is involved in early events necessary for chlamydiae to gain entry and-or establish an intracellular replication niche. Unlike, TarP, TepP, and CT694, ectopic expression of CT695 in yeast did not result in an overt phenotype that would give hints with regard to function [53]. The apparent localization of CT695 adjacent to the inclusion membrane is interesting. CT695 does not contain predicted trans-membrane domains and may associate with membranes through interactions with other proteins or via direct association with lipids. CT694 contains a membrane localization domain found in effectors such as *Yersinia* YopE and *Pseudomonas* ExoS [54]. It is therefore possible that CT695 could associate with membranes via a similar mechanism. Regardless, our immunolocalization studies imply that CT695 is likely a multifunctional effector necessary at multiple stages of chlamydial development.

While the BlaM reporter system does not provide information regarding effector localization, there are several advantages to using this approach. *Chlamydia* employ T2S, T3S, and T5S to deploy host-interactive proteins and estimates based on current findings suggest as many as 80 proteins in the chlamydial secretome [55]. Therefore, there is certainly a need for an approach to screen for secreted proteins in the context of a chlamydial infection. Although we used fixed samples for microscopy, secretion can easily be visualized in live cells using this reporter [26]. This opens numerous possibilities that include quantitative and kinetic studies of effector secretion and translocation [56]. In addition, the BlaM reporter system has been employed during animal infection studies to discriminate cell types susceptible to effector injection [57] or separate infected from bystander cells [58]. This system would also provide an efficacious platform to study the nature of T3 secretion signals. All of these approaches are adaptable for the study of *Chlamydia* pathogenesis. We conclude that use of BlaM fusion constructs will prove to be an efficacious approach for the study of protein secretion by chlamydiae.

## Supporting Information

S1 Fig Progeny IFU counts for *C*. *trachomatis* strains. Triplicate cultures of HeLa cells were infected at an MOI of 1 with untransformed *C*. *trachomatis* (L2), *C*. *trachomatis* expressing pL2Dest (Vector), or *C*. *trachomatis* expressing individual BlaM-fusions. Cultures were harvested at 24 hpi and lysates were plated onto fresh HeLa monolayers. Progeny IFU counts were enumerated at 24 hpi. Mean progeny counts are shown and error bars represent one standard deviation. No statistically significant differences were observed.
(TIF)

S1 Table Primers used in plasmid generation. (DOCX)

S2 Table Gene expression primers. (DOC)
