# Supplementary figures and images for: Application of β-Lactamase Reporter Fusions as an Indicator of Effector Protein Secretion during Infections with the Obligate Intracellular Pathogen Chlamydia trachomatis
Source: PLoS One. 2015 Aug 10;10(8):e0135295. doi: 10.1371/journal.pone.0135295 (PMC4530969; doi:10.1371/journal.pone.0135295)

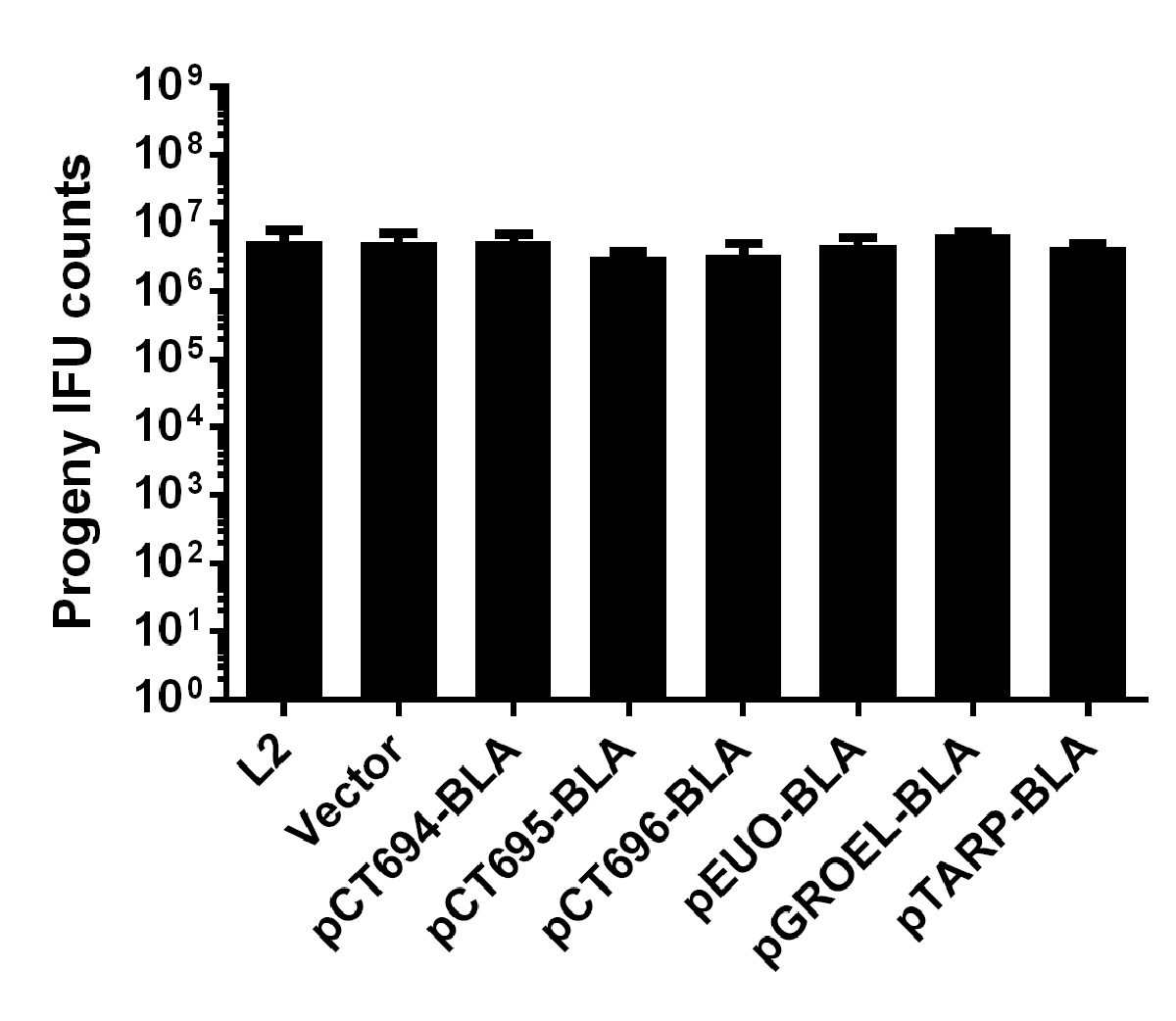

Supplement: S1 Fig — Triplicate cultures of HeLa cells were infected at an MOI of 1 with untransformed C. trachomatis (L2), C. trachomatis expressing pL2Dest (Vector), or C. trachomatis expressing individual BlaM-fusions. Cultures were harvested at 24 hpi and lysates were plated onto fresh HeLa monolayers. Progeny IFU counts were enumerated at 24 hpi. Mean progeny counts are shown and error bars represent one standard deviation. No statistically significant differences were observed. (TIF) [file pone.0135295.s001.tif]
