# Supplementary material for: Application of β-Lactamase Reporter Fusions as an Indicator of Effector Protein Secretion during Infections with the Obligate Intracellular Pathogen Chlamydia trachomatis
Source: PLoS One. 2015 Aug 10;10(8):e0135295. doi: 10.1371/journal.pone.0135295 (PMC4530969; doi:10.1371/journal.pone.0135295)
Supplement: S1 Table — (DOCX) [file pone.0135295.s002.docx]

**Table S1**. Primers^a^ used in plasmid generation

| **pL2dest^b^** | | | | |
| --- | --- | --- | --- | --- |
| mC@GFP F | | | GATATGGTGGATCCCCGGGTACCAATGGTGAGCAAGGGCGAGGAGG | |
| mC@GFP R | | | CTGCCACTCATCGCAGTACTGTTGTAACCTCTACAAATGTGGTATGGCTG | |
| SalI+AscI+Chlor F | | | GGGGTCGACGGCGCGCCCTGTGACGGAAGATCACTTCGCAGAATAAA | |
| SalI+AscI+Chlor R | | | GGGGTCGACGGCGCGCCCACCAGGCGTTTAAGGGCACCAATA | |
|  | | |  | |
| **pUCNmp^c^** | | | | |
| NmP@pUC F | | GTCTCCGGGAGCTGCATGTGGCCTGCAGATGCCCGACGG | | |
| NmP@pUC R | | CCCCGAAAAGTGCCACCTGACGGGGATCCACCATATCGGCTTCC | | |
|  | |  | | |
| **pUCNmp cloning^d^** | | | | |
| 694@bla F | GGAAGCCGATATGGTGGATCCCCATGAGTATTCGACCTACTAATGGGAGTGG | | | |
| 694@bla R | GGGCGACACGGAAATGTTGAATACTCATGTCTAAGAAAACAGAAGAAGTTATGACAGTTAG | | | |
| 695@bla F | GGAAGCCGATATGGTGGATCCCCATGAGTAGCATAAGCCCTATAGGGGG | | | |
| 695@bla R | GGGCGACACGGAAATGTTGAATACTCATGATATTCCCAACCGAAGAAGGATC | | | |
| 696@bla F | GGAAGCCGATATGGTGGATCCCCATGCTATTAGATTCTCGTTTCCCTACAGA | | | |
| 696@bla R | GGGCGACACGGAAATGTTGAATACTCATACGAGCTTCCTTACGGAAAGTTCC | | | |
| euo@bla F | GGAAGCCGATATGGTGGATCCCCATGGAATGCTTACAACAAGATACAG | | | |
| euo@bla R | GGGCGACACGGAAATGTTGAATACTCATTGAGATAAAATTTTCTGCGTCTGCC | | | |
| groEL@bla F | GGAAGCCGATATGGTGGATCCCCATGCCTCACGACAACAATGAGATGC | | | |
| groEL@bla R | GGGCGACACGGAAATGTTGAATACTCATAGTTTTTTTCTCTTTTGCTGCAGGAG | | | |
| tarp@bla F | GGAAGCCGATATGGTGGATCCCCATGACGAATTCTATATCAGGTGATC | | | |
| tarp@bla R | GGGCGACACGGAAATGTTGAATACTCATTCCTACGGTATCAATCAGTGAGC | | | |
|  |  | | | |
| **Fusion transfer^e^** | | | | |
| NmP+BlaFus+AscI F | | | | GGGGGCGCGCCGCCTGCAGATGCCCGACGG |
| NmP+BlaFus+AscI R | | | | GGGGGCGCGCCGAGTAAACTTGGTCTGACAGTTACCAATGC |

^a^ All primers are listed 5’ to 3’

^b^ Primers used for alteration of pGFP::SW2 into pL2dest

^c^ Primers for construction of pUCNmp

^d^ Primers used to insert respective chlamydial genes into pUCNmp

^e^ Primer set used to transfer blaM-fusion construct from pUCNmp into pL2dest
