# Supplementary material for: Application of β-Lactamase Reporter Fusions as an Indicator of Effector Protein Secretion during Infections with the Obligate Intracellular Pathogen Chlamydia trachomatis
Source: PLoS One. 2015 Aug 10;10(8):e0135295. doi: 10.1371/journal.pone.0135295 (PMC4530969; doi:10.1371/journal.pone.0135295)
Supplement: S2 Table — (DOC) [file pone.0135295.s003.doc]

**Table S2**. Gene expression primers^a^

| **Genomic chlamydial genes^b^** | |
| --- | --- |
| rpoD s | GCGGTGTTTCCATTGTCGTCATA |
| rpoD α-s | ATTTCTCTCAGCTCGCGCTTTC |
| CT694 s | AACCTATCTGTGGGAGGGAAGCAT |
| CT694 α-s | TTGCCCTGAACCAGGACTAGAGAA |
| CT695 s | ATAGATCACTTGGCGGAGAAAGCC |
| CT695 α-s | GCTGCTCATTCACAGGAGGGAATA |
| CT696 s | CGCGTAGTCACGAGGCAATTAAGA |
| CT696 α-s | TCGGTAATCACGCCTCCGATAAAC |
| 16s s | CCTGGTAGTCCTTGCCGTAAAC |
| 16s α-s | TACTCCTCAGGCGGCATACTTA |
|  |  |
| **Bridge primers^c^** | |
| CT694-5 s | ATGAGTATTCGACCTACTAATGGG |
| CT694-5 α-s | GATATTCCCAACCGAAGAAGGATC |
| CT695-6 s | GTGAGTAGCATAAGCCCTATAGG |
| CT695-6 α-s | ACGAGCTTCCTTACGGAAAG |
|  |  |
| **Vector-encoded genes^d^** | |
| mCherry s | AGATCAAGCAGAGGCTGAAGCTGA |
| mCherry α-s | ACTGTTCCACGATGGTGTAGTCCT |
| CT694-blaM s | TCCAAACACTAACTGTCATAACTTCTTCTG |
| CT695-blaM s | TGCGTATGATCGAAGGTTTGGTAAC |
| CT696-blaM s | CCTCGGAATCAGGAGATGGAACT |
| euo-blaM s | GTCTTGCAGCCATGAGAGAAGG |
| groEL-blaM s | TTCCCTGCATACCGCTGTAGAT |
| tarp-blaM s | CAAGCAGCAACCCAAACAACTC |
| fus-blaM α-s | GGAATAAGGGCGACACGGAAAT |

^a^ All primers sequences are given 5’ to 3’

^b^ Primers used for qRT-PCR analysis of endogenous gene expression.

^c^ Primers that bridge adjacent genes to detect co-transcription.

^d^ Primers used to assess transcript levels of pL2dest-encoded genes.
